# Impact of the COVID-19 pandemic on healthcare workers risk of infection and outcomes in a large, integrated health system.

**DOI:** 10.21203/rs.3.rs-61235/v1

**Published:** 2020-08-19

**Authors:** Anita D Misra-Hebert, Lara Jehi, Xinge Ji, Amy S. Nowacki, Steven Gordon, Paul Terpeluk, Mina K. Chung, Reena Mehra, Katherine M. Dell, Nathan Pennell, Aaron Hamilton, Alex Milinovich, Michael W. Kattan, James B. Young

**Affiliations:** Cleveland Clinic; Cleveland Clinic; Cleveland Clinic; Cleveland Clinic; Cleveland Clinic; Cleveland Clinic; Cleveland Clinic; Cleveland Clinic; Cleveland Clinic Children’s Hospital; Cleveland Clinic; Cleveland Clinic; Cleveland Clinic; Cleveland Clinic; Cleveland Clinic

**Keywords:** SARS Coronavirus, Healthcare Workers, Employee Health

## Abstract

**Background:**

Understanding the impact of the COVID-19 pandemic on healthcare workers (HCW) is crucial.

**Objective:**

Utilizing a health system COVID-19 research registry, we assessed HCW risk for COVID-19 infection, hospitalization and intensive care unit (ICU) admission.

**Design:**

Retrospective cohort study with overlap propensity score weighting.

**Participants:**

Individuals tested for SARS-CoV-2 infection in a large academic healthcare system (N=72,909) from March 8-June 9 2020 stratified by HCW and patient-facing status.

**Main Measures:**

SARS-CoV-2 test result, hospitalization, and ICU admission for COVID-19 infection.

**Key Results:**

Of 72,909 individuals tested, 9.0% (551) of 6,145 HCW tested positive for SARS-CoV-2 compared to 6.5% (4353) of 66,764 non-HCW. The HCW were younger than non-HCW (median age 39.7 vs. 57.5, p<0.001) with more females (proportion of males 21.5 vs. 44.9%, p<0.001), higher reporting of COVID-19 exposure (72 vs. 17 %, p<0.001) and fewer comorbidities. However, the overlap propensity score weighted proportions were 8.9 vs. 7.7 for HCW vs. non-HCW having a positive test with weighted odds ratio (OR) 1.17, 95% confidence interval (CI) 0.99–1.38. Among those testing positive, weighted proportions for hospitalization were 7.4 vs.15.9 for HCW vs. non-HCW with OR of 0.42 (CI 0.26–0.66) and for ICU admission: 2.2 vs.4.5 for HCW vs. non-HCW with OR of 0.48 (CI 0.20–1.04). Those HCW identified as patient-facing compared to not had increased odds of a positive SARS-CoV-2 test (OR 1.60, CI 1.08–2.39, proportions 8.6 vs. 5.5), but no statistically significant increase in hospitalization (OR 0.88, CI 0.20–3.66, proportions 10.2 vs. 11.4) and ICU admission (OR 0.34, CI 0.01–3.97, proportions 1.8 vs. 5.2).

**Conclusions:**

In a large healthcare system, HCW had similar odds for testing SARS-CoV-2 positive, but lower odds of hospitalization compared to non-HCW. Patient-facing HCW had higher odds of a positive test. These results are key to understanding HCW risk mitigation during the COVID-19 pandemic.

## Introduction

Understanding the risks associated with the COVID-19 pandemic^1^ on healthcare workers (HCW), including the risk of acquisition at work vs other settings, is crucial. Prediction of risk can inform how to protect HCWs such as recommendations on use of personal protective equipment (PPE) at work or in the community. The presence of specific symptoms in HCW (China, US)^2,3^ and symptoms predicting SARS-CoV-2 test positivity in HCW (Netherlands)^4^ have been reported as well as characteristics associated with HCW deaths (China).^5^ Based upon data from the 2018 National Health Interview Survey, it was estimated that 26.6% of patient facing HCW were at increased risk for poor outcomes from COVID-19 infection because of their comorbidities or age.^6^ Reported experiences in China^7^, Italy^8^ and Solano County, CA without initial use of PPE^9^ showed higher percentages of HCW testing positive for COVID-19. In contrast, a screening study of HCW in England showed no significant difference in positive results between clinical and nonclinical staff with implementation of isolation and PPE protocols perhaps suggesting predominant community rather than nosocomial transmission patterns.^10^ The extent of risk modification with PPE remains unclear.^7–9,11^ A recent prospective study in the United Kingdom and US suggested a five-fold increased risk for HCW caring for patients with COVID-19 compared to HCW not caring for patients with COVID-19, even with the use of PPE^12^ while another study of HCW in a large healthcare system showed a decrease in positive tests for SARS-CoV-2 associated with a universal masking recommendation.^13^ This heterogeneous landscape makes it difficult for the HCW community to determine actual risk of acquiring COVID-19 in healthcare vs. community settings and the effectiveness of various risk-mitigating strategies.

The Cleveland Clinic Health System (CCHS) is a large, integrated health system with 55,574 eligible employees in Ohio & Florida. The CCHS initiated multiple COVID-19 related public health initiatives to mitigate the spread of the disease and its impact on the HCW community. In parallel, we maintained a rigorous, comprehensive, and prospective registry capturing disease risk and progression in all individuals tested for COVID-19 in our health system. In this study, we aimed to assess whether HCW are at higher risk for COVID-19 infection, COVID-19 related hospitalization, and intensive care unit (ICU) admission compared to non-HCW using advanced statistical methodology to account for various confounders.

## Methods

### Cohort definition

#### COVID-19 Cleveland clinic enterprise registry.

All patients, regardless of age, who were tested for COVID-19 at all CCHS locations in Ohio and Florida were included in this research registry. For this study, all individuals who were tested for COVID-19 in the CCHS between March 8, 2020 and June 9, 2020 were studied. This registry provides better representation of the overall population than testing restricted to one geographic health system site. Registry variables were chosen to reflect available literature on COVID-19 disease characterization, progression, and proposed treatments, including medications initially thought to have potential for benefit after drug-repurposing network analysis.^14^ Capture of detailed research data was facilitated by the creation of standardized clinical templates implemented across the healthcare system as patients were seeking care for COVID-19-related concerns. Data were extracted via previously validated automated feeds from electronic health records^15^ (EPIC; EPIC Systems Corporation) and manually by a study team trained on uniform sources for the study variables. Study data were collected and managed using REDCap electronic data capture tools hosted at the Cleveland Clinic.^16,17^ The COVID-19 Research Registry team includes a “Reviewer” group and a “Quality Assurance” group. The reviewers were responsible for manually abstracting and entering a subset of variables that cannot be automatically extracted from the electronic health record (EHR). Reviewers were also asked to verify high-priority variables that have been automatically pulled into the database from EPIC. The Cleveland Clinic Institutional Review Board approved this study and waived the requirements for written informed consent.

#### Identification o f HCW.

Individuals were identified as HCW through CCHS Occupational Health and their job description was identified as having direct contact with patients or “patient-facing” vs. non-patient facing based upon the listing in the CCHS Human Resources database.

### Public health and employer-initiated risk mitigation measures

Public health guidelines for CCHS employees and availability of testing for COVID-19 changed rapidly between 3/6/2020–4/24/2020 (Appendix 1), the most relevant being the *recommendation* for universal masking for CCHS employees on 4/7/2020 and *requirement* on 4/24/2020. Regarding state public health orders, a stay at home order was issued in Ohio on 3/22/2020 with phased re-opening in May starting with restaurants and bars on 5/14/2020^18^ and in Florida a public health advisory was issued on 3/25/2020 addressing vulnerable populations, private gatherings, and workforce density^19^ with reopening beginning on 5/18/2020.^20^

### Statistical Analysis

All descriptive statistics were reported as counts (percentages) or median (interquartile ranges [IQRs]). For comparison of demographic variables and comorbidities among cohorts, Wilcoxon signed-rank tests were used for numeric variables, while χ^2^ or Fisher exact tests were used for categorical variables. To address differences in baseline characteristics of non-HCW and HCW, specifically as related to underlying comorbidities, and the limitations of current literature that failed to account for such differences, we leveraged appropriate statistical methodology to study our research questions. Overlap propensity score^21,22^ weighting was performed to address potential confounding in comparing HCW to non-HCW given their baseline differences. The overlap propensity score weighting method was chosen given its benefits of preservation of numbers of individuals in each group and of achieving higher levels of precision in the resulting estimates. This methodology is preferred when the propensity score distributions among the groups are dissimilar and when the propensity scores are clustered near the extremes (i.e. close to zero or one). A propensity score for being a HCW was estimated from a multivariable logistic regression model. For the outcome of being test positive for COVID-19, the propensity score logistic regression model included covariates that were found to be associated with a positive COVID-19 test outcome in our previous work.^23^ For the outcomes of hospital and intensive care unit (ICU) admission of COVID-19 test positive patients, the propensity score covariates are those that were found associated with COVID-19 hospitalization outcome in our previous work including age, race, ethnicity, gender, smoking history, body mass index, median income, population per housing unit, presenting symptoms (including fever, fatigue, shortness of breath, diarrhea, vomiting), comorbidities (including asthma, hypertension, diabetes, immunosuppressive disease), medications (including immunosuppressive treatment, non-steroidal anti-inflammatory drugs [NSAIDs]), and laboratory values (including pre-testing platelets, aspartate aminotransferase, blood urea nitrogen, chloride, and potassium).

The overlap propensity score weighting method was then applied where each patient’s statistical weight is the probability of that patient being assigned to the opposite group.^21^ Overlap propensity score weighted logistic regression models were used to investigate associations between HCW status and the probability of testing positive for SARS-CoV-2, hospital admission for COVID-19 and ICU admission for COVID-19 illness. The results are thus reported as weighted proportions, odds ratios and 95% confidence intervals. All statistical analyses were performed using R 3.5 and SAS version 9.4 (SAS Institute). *P* values were 2-sided, with a significance threshold of .05.

We then used locally weighted regression smoother (LOESS) to summarize the trend of COVID-19 test positivity through the study period for HCW and non-HCW as related to the public health measures instituted at the state level in Ohio and those specific to the CCHS.

## Results

### 

#### Overall tested cohort characteristics.

Of the 72,909 individuals tested for COVID-19 in the CCHS Research Registry, there were 6,145 HCW and 66,764 non-HCW with over 90% of HCW and 75% of non-HCW tested from Ohio. There were 9% of HCW who tested positive for COVID-19 compared to 6.5% of non-HCW, p<0.001 (Table 1). The HCW tested were younger than non-HCW (median age 39.7 vs. 57.5, p<0.001) with more females (proportion of males 21.5 vs. 44.9%, p<0.001), higher proportion of Asian and lower proportion of Black persons (3.4 vs. 1.0% and 16.2 vs. 18.3%, respectively, p<0.001), higher proportion identifying as non-Hispanic (90.8 vs. 87.6%, p <0.001), higher median income, and higher proportion of non-smokers. The neighborhood characteristics of population density as measured per square kilometer was similar for tested HCW vs. non- HCW while the population per housing unit was slightly higher. The HCW were more likely to report an exposure to COVID-19 (72.0% vs. 17.0%, p<0.001) and also to report having a family member with COVID-19 (28.3 vs. 14.2%, p 0.005). Regarding presenting symptoms, a slightly higher proportion of HCW reported cough (32.0 vs. 29.6%, p 0.001), a lower proportion reported fever (15.0 vs. 19.5%, p <0.001) or shortness of breath (14.6 vs. 25.7%, p<0.001), while a higher proportion reported diarrhea (11.9 vs. 9.5%, p<0.001) and lower proportion reported vomiting (7.4 vs. 9.7%, p <0.001). Of note, the tested HCW were, in general, healthier than the non-HCW group. The HCW had a lower proportion of several comorbidities including chronic obstructive pulmonary disease(COPD)/emphysema, diabetes, hypertension, coronary artery disease, heart failure, cancer, history of transplant, or immunosuppressive disease and were more likely to have received the influenza vaccine (85.9 vs. 45.4%, p <0.001). The HCW tested had a lower proportion of previous prescriptions for immunosuppressive treatment, NSAIDs, steroids, carvedilol, angiotensin converting enzyme inhibitors, angiotensin receptor blockers, or melatonin.

#### COVID-19 cohort characteristics and outcomes:

There were 551 HCW and 4,353 non-HCW who tested positive for COVID-19 (Appendix Table 2). Of those who tested positive for COVID-19, a lower proportion of HCW were hospitalized compared to non-HCW (38 or 6.9% HCW vs. 1205 or 27.7% non-HCW) or were admitted to the intensive care unit (10 or 1.8% HCW vs. 470 or 10.8% non-HCW). In the group who tested positive for COVID-19, there was a greater proportion of HCW of Asian and White race compared to non-HCW (2.9 vs. 0.8% and 61.0 vs 56.4%, respectively), a similar proportion of HCW with a positive COVID-19 test had presenting symptoms of cough, fatigue, diarrhea, loss of appetite, and vomiting and a lower proportion had fever or shortness of breath. Lower proportions of HCW testing positive had COPD/emphysema, diabetes, coronary artery disease, heart failure, cancer, or immunosuppressive disease and were previously prescribed carvedilol, angiotensin converting enzyme inhibitors, angiotensin receptor blockers or melatonin compared to non-HCW. The neighborhood population characteristics of population density or population per housing unit did not differ for those HCW who tested positive and median income was slightly higher compared to non-HCW.

#### Overlap propensity weighting:

**Figure 1:**
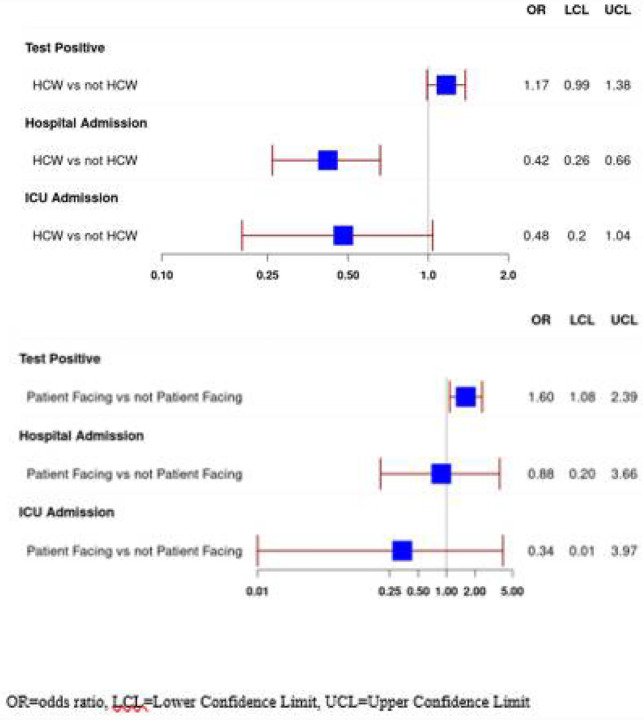
(Top)a: Odds of SARS-CoV-2 positive test, Hospital Admission if SARS-CoV-2 positive, and Intensive Care Admission if SARS-CoV-2 positive by Healthcare Worker (HCW) Status (Bottom)b: Odds of SARS-CoV-2 positive test, Hospital Admission if SARS-CoV-2 positive, and Intensive Care Admission if SARS-CoV-2 positive by Patient-Facing and Non-Patient Facing Healthcare Worker Status

Using the variables in the prediction model for COVID-19 test positivity,^23^ overlap propensity score weighting (Table 2) resulted in propensity score weighted proportions of 7.7 vs. 8.9 for non-HCW vs. HCW having a positive test and produced an overlap propensity score weighted odds ratio of 1.17 with a 95% confidence interval (CI) of 0.99–1.38 for a HCW having a positive test compared to a non-HCW (Figure 1 a). Then using the variables which predicted hospitalization for COVID-19 infection, overlap propensity score weighting was applied (Table 3) with weighted proportions for being hospitalized 15.9 vs. 7.4 for non-HCW vs. HCW, an odds ratio of 0.42 (CI 0.26 −0.66) for a HCW being hospitalized for COVID-19 compared to a non-HCW. For ICU admission, weighted proportions were 4.5 vs.2.2 for non-HCW vs. HCW with an odds ratio of 0.48 (CI 0.20–1.04) for HCW being admitted to the ICU compared to non-HCW (Figure 1 a).

### Subgroup analysis

We then compared characteristics of HCW identified as having positions that required direct contact with patients (“patient facing”) and those that did not. There were 5,159 HCW with patient-facing positions and 986 HCW in non-patient facing roles (Appendix Table 3). The HCW with patient-facing roles were younger (median age 38 vs. 47 years, p<0.001), with more females (proportion males 20.6 vs. 26.2%, p <0.001), lower proportion of Black race and higher Asian race, and with greater proportion reporting exposure to COVID-19 (73.7 vs. 62.9%, p < 0.001). The patient-facing HCW had lower proportions presenting with fatigue or shortness of breath and higher proportion with loss of appetite. There were no significant differences in laboratory values upon presentation. The patient-facing HCW had lower proportions of some previously prescribed medications including NSAIDs, steroids, angiotensin converting enzyme inhibitors, angiotensin receptor blockers, and melatonin. The patient-facing HCW group had lower proportions of comorbidities including COPD/emphysema, diabetes, hypertension, coronary artery disease, cancer, connective tissue disease, and immunosuppressive disease. Applying the overlap propensity score weighting (Appendix Tables 4, 5; Figure 1 b) showed patient-facing HCW with increased odds of having a positive SARS-CoV-2 test result (OR 1.60, CI 1.08–2.39, weighted proportions 8.6 vs. 5.5), and lower but non-significant odds of hospital admission (OR 0.88, CI 0.20–3.66, proportions10.2 vs. 11.4) and ICU admission (OR 0.34, CI 0.01–3.97, proportions 1.8 vs. 5.2).

### Temporal relationship between disease prevention measures and positive tests

**Figure 2:**
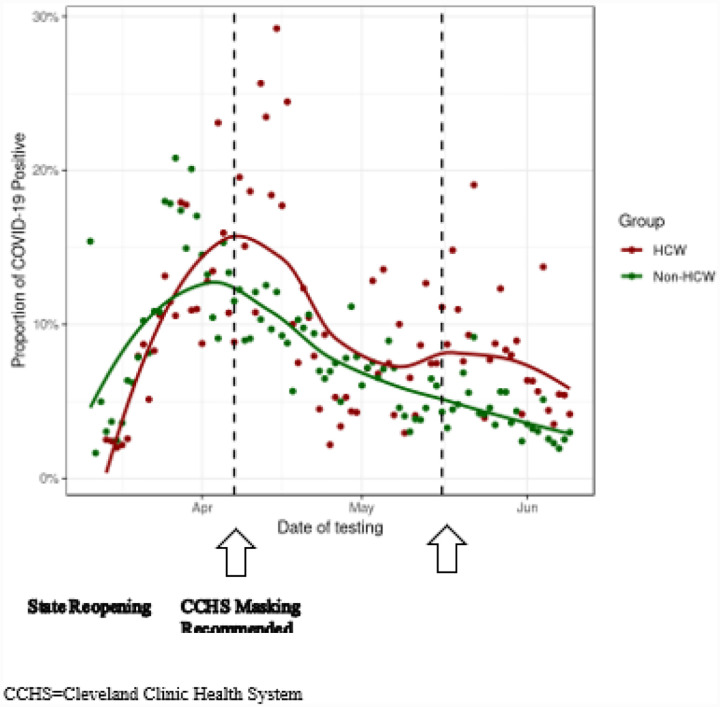
Proportion of SARS-CoV-2 Positive Results During Study Period

The summary of the trend of SARS-CoV-2 positive test results in the study period is shown in Figure 2. The overall proportion of positive COVID-19 test results decreased during the study period and the trend for HCW and followed that of non-HCW.

## Discussion

Our analysis of HCW compared to non-HCW who were tested for SARS-CoV-2 in one health system with 2 geographic locations (Ohio, Florida), and which controlled for significant differences in baseline characteristics between the HCW and non-HCW groups, showed that the odds of having a positive COVID-19 test were not significantly different for HCW compared to non-HCW, and HCW had lower odds of subsequent hospitalization, and without statistically significant differences in ICU admission compared to non-HCW once they tested positive. The HCW classified as having patient-facing positions had higher and significant odds of a positive COVID-19 test with insignificant differences detected compared to non-patient facing HCW in outcomes of hospitalization or ICU admission. We found a similar proportion of HCW with a positive COVID-19 test had presenting symptoms of cough, fatigue, diarrhea, loss of appetite, and vomiting while a lower proportion had fever or shortness of breath. We note that we were not able to capture the symptoms of loss of taste and/or smell and that these symptoms may be common especially with mild cases of COVID-19.^24,25^

The overall proportion of COVID-19 positive tests in HCW was low and decreased during the study period corresponding with implementation of risk-mitigation measures in our health system such as the recommendations for universal masking and physical distancing but also followed the trend for non-HCW. Several of the previous studies of HCW risk for infection during the COVID-19 pandemic were limited by their sample sizes, 7–9 lack of generalizability for healthcare systems that have adequate access to PPE,^7–9^ methodology relying on self-report,^12^ limited ability to adjust for known risk factors of disease susceptibility and progression^7–10,12^ and lacking data to investigate the relative effects of dual exposure of HCW to COVID-19 in the community versus the workplace.^7–10,12^ The fact that HCW identified as patient-facing had a significantly higher odds for SARS-CoV-2 test positivity suggests an increased risk of COVID-19 infection with work exposure. However, it is important to note in our study that that over 70% of the HCW group reported an exposure to COVID-19 with 28% reporting exposure to a family member with COVID-19. In our study, we were not able to confirm if the patient-facing HCW were working in patient-facing areas the 14-day period before the test was ordered when exposure could have occurred, or whether the exposure occurred with or without PPE - both in the workplace or in the community, or the relative contribution of initially prioritizing testing availability to HCW with reported exposures. While the risk to HCW attributed to community spread may not be captured in our available data, the reported exposure risk including the higher proportion of HCW vs. non-HCW reporting exposure to a family member with COVID-19 suggests a degree of community acquisition of infection. A potential contributing factor to community acquisition is that HCWs, particularly patient-facing HCW, are less able to follow stay-at-home guidelines or work remotely from home. Indeed, while PPE use is associated with decrease risk of infection from coronavirus,^26^ a recent report estimated less than 5% risk to HCW inadvertently exposed to patients not known to be SARS-CoV-2-positive at the time of initial exposure with exposure likely occurring without appropriate PPE^27^ suggesting that the work exposure risk may actually be low. However, universal pandemic precautions have been recommended for optimal risk mitigation for HCW.^28^

## Conclusion

In our analysis of one healthcare system which implemented significant risk mitigation strategies to prevent the spread of COVID-19 infection, and which controlled for significant baseline differences in HCW compared to non-HCW, the odds for SARS-CoV-2 infection were similar for HCW and non-HCW and HCW had lower odds for COVID-19 related hospitalization. The patient facing HCW had higher odds of SARS-CoV-2 infection.

## Supplementary Material

Supplement

## Figures and Tables

**Table 1:** Characteristics of All Patients Tested for SARS-CoV-2 by Health Care Worker (HCW) Status

	Non-HCW Number(%) or MediantInterquaitile Range]	HCW Number(%) or Mediantlnterquartile Range]	p-value
**Number**	66764	6145	
**SARS-CoV-2 Positive**	4353 ( 6.5)	551 ( 9.0)	<0.001
**Demographics:**			
Location			<0.001
Ohio	50204 ( 75.2)	5642 ( 91.8)	
Florida	13957 ( 20.9)	503 ( 8.2)	
Unknown	2603 ( 3.9)	0	
Race			<0.001
Asian	672 ( 1.0)	206 ( 3.4)	
Black	12201 ( 18.3)	995 ( 16.2)	
Other	5394 ( 8.1)	490 ( 8.0)	
White	48497 ( 72.6)	4454 ( 72.5)	
Male	29959 ( 44.9)	1322 ( 21.5)	<0.001
Non-Hispanic	58496 ( 87.6)	5577 ( 90.8)	<0.001
Smoking			<0.001
Current Smoker	9316 ( 14.0)	147 ( 2.4)	
Former Smoker	30196 ( 45.2)	1737 ( 28.3)	
No	27048 ( 40.5)	4259 ( 69.3)	
Unknown	204 ( 0.3)	2 ( 0.0)	
Age	57.53 [39.32, 70.26]	39.67 [31.29, 51.80]	<0.001
**Exposure history:**			
Exposed to COVID-19	11369 ( 17.0)	4424 ( 72.0)	<0.001
Family member with COVID-19	9503 ( 14.2)	1740 (28.3)	<0.001
**Presenting symptoms:**			
Cough	19744 ( 29.6)	1968 ( 32.0)	0.001
Fever	12997 ( 19.5)	922 ( 15.0)	<0.001
Fatigue	8020 ( 12.0)	637 ( 10.4)	<0.001
Sputum production	402 ( 0.6)	45 ( 0.7)	0.244
Flu-like symptoms	5949 ( 8.9)	423 ( 6.9)	<0.001
Shortness of breath	17133 ( 25.7)	898 ( 14.6)	<0.001
Diarrhea	6335 ( 9.5)	731 ( 11.9)	<0.001
Loss of appetite	1505 ( 2.3)	299 ( 4.9)	<0.001
Vomiting	6471 ( 9.7)	454 ( 7.4)	<0.001
**Co-morbidities:**			
Body Mass Index	28.37 [25.85, 31.07]	28.37 [27.37, 29.13]	<0.001
Chronic Obstructive Pulmonary Disease/emphysema	8247 ( 12.4)	166 ( 2.7)	<0.001
Asthma	12057 ( 18.1)	1343 ( 21.9)	<0.001
Diabetes	13418 ( 20.1)	428 ( 7.0)	<0.001
Hypertension	30727 ( 46.0)	1505 ( 24.5)	<0.001
Coronary artery disease	10181 ( 15.2)	204 ( 3.3)	<0.001
Heart failure	8192 ( 12.3)	93 ( 1.5)	<0.001
Cancer	12469 ( 18.7)	646 ( 10.5)	<0.001
Transplant history	1120 ( 1.7)	19 ( 0.3)	<0.001
Multiple sclerosis	493 ( 0.7)	43 ( 0.7)	0.794
Connective tissue disease	2376 ( 3.6)	178 ( 2.9)	0.008
Inflammatory Bowel Disease	1604 ( 2.4)	112 ( 1.8)	0.005
Immunosuppressive disease	10375 ( 15.5)	414 ( 6.7)	<0.001
**Vaccination history:**			
Influenza vaccine	30340 ( 45.4)	5277 ( 85.9)	<0.001
Pneumococcal polysaccharide vaccine	17808 ( 26.7)	451 ( 7.3)	<0.001
**Laboratory findings upon presentation:**			
Pre-testing platelets	238.00 [238.00, 238.00]	238.00 [238.00, 238.00]	0.137
Pre- testing AST	23.00 [23.00, 23.00]	23.00 [23.00, 23.00]	0.051
Pre- testing BUN	16.00 [16.00, 16.00]	16.00 [16.00, 16.00]	<0.001
Pre- testing Chloride	101.00 [101.00, 101.00]	101.00 [101.00, 101.00]	0.092
Pre- testing Creatinine	0.92 [0.92, 0.92]	0.92 [0.92, 0.92]	<0.001
Pre-testing hematocrit	39.50 [39.50, 39.50]	39.50 [39.50, 39.50]	0.41
Pre- testing Potassium	4.10 [4.10, 4.10]	4.10 [4.10, 4.10]	0.081
**Home medications:**			
Immunosuppressive treatment	2392 (3.6)	270 ( 4.4)	<0.001
Nonsteroidal Anti-inflammatory Drugs	19651 ( 29.4)	1048 ( 17.1)	<0.001
Steroids	11838 ( 17.7)	969 ( 15.8)	<0.001
Carvedilol	2803 ( 4.2)	40 ( 0.7)	<0.001
Angiotensin converting enzyme inhibitor	7568 ( 11.3)	329 ( 5.4)	<0.001
Angiotensin receptor blocker	5698 ( 8.5)	228 ( 3.7)	<0.001
Melatonin	3078 ( 4.6)	52 ( 0.8)	<0.001
**Social influencers of health:**			
Population Per Square Kilometer	3.03 [2.63, 3.29]	3.01 [2.64, 3.28]	0.568
Median Income per $1000	58.09 [41.62, 76.21]	64.78 [49.78, 85.54]	<0.001
Population Per Housing Unit	2.22 [1.93, 2.49]	2.26 [2.01, 2.51]	<0.001

**Table 2:** All tested patients: Overlap Propensity Score-Weighted Characteristics a a Reported are either weighted proportions (for categorical variables) or weighted means (for numeric variables)

	Non- HCW	HCW
Count	66764	6145
Race		
Asian	2.3	2.3
Black	17.9	17.9
Other	8	8
White	71.8	71.8
Male	25.1	25.1
Non-Hispanic	90.2	90.2
Smoking		
Current Smoker	3.7	3.7
Former Smoker	31.7	31.7
No	64.6	64.6
Unknown	0	0
Age	43.38	43.38
Exposed to COVID-19	58.1	58.1
Family member with COVID-19	0.1	0.1
Cough	34.4	34.4
Fever	18	18
Fatigue	11.7	11.7
Sputum production	0.9	0.9
Flu-like symptoms	7.3	7.3
Diarrhea	12.7	12.7
Loss of appetite	4.3	4.3
Vomiting	8.7	8.7
Asthma	22.9	22.9
Coronary artery disease	4.6	4.6
Transplant history	0.5	0.5
Connective tissue disease	3.4	3.4
Inflammatory Bowel Disease	2.1	2.1
Influenza vaccine	79.2	79.2
Pneumococcal polysaccharide vaccine	11.3	11.3
Pre-testing platelets	240.05	240.05
Pre- testing Aspartate Aminotransferase	23.87	23.87
Pre- testing Chloride	101.05	101.05
Pre- testing Creatinine	0.92	0.92
Pre-testing Hematocrit	39.48	39.48
Pre- testing Potassium	4.09	4.09
Nonsteroidal Anti-inflammatory Drugs	20.4	20.4
Steroids	17.4	17.4
Carvedilol	0.9	0.9
Angiotensin Converting Enzyme inhibitor	6.7	6.7
Angiotensin Receptor Blocker	4.9	4.9
Melatonin	1.2	1.2
Population Per Square Kilometer	2.92	2.92
Median Income (thousands of dollars)	68.20	68.20
Body Mass Index	2.92	2.92
**Final Result = Positive Test for SARS-CoV-2**	7.7	8.9
**Odds Ratio (95% Confidence Interval)**		1.17 (0.99, 1.38)

**Table 3:** All test positive patients: Overlap Propensity Score–Weighted Characteristics^a^

	Non-HCW	HCW
Count	4353	551
Race		
Asian	2.1	2.1
Black	28.6	28.6
Other	9.4	9.4
White	59.9	59.9
Male	30.7	30.7
Ethnicity		
Hispanic	3.3	3.3
Non-Hispanic	90.2	90.2
Unknown	6.4	6.4
Smoking		
Current Smoker	2.9	2.9
Former Smoker	18.6	18.6
No	73.7	73.7
Unknown	4.8	4.8
Age	43.50	43.50
Fever	44.8	44.8
Fatigue	55.2	55.2
Shortness of breath	37	37
Diarrhea	35	35
Vomiting	24.7	24.7
Asthma	12.6	12.6
Diabetes	10.4	10.4
Hypertension	22.2	22.2
Immunosuppressive treatment	6.9	6.9
Immunosuppressive disease	4.5	4.5
Pre-testing platelets	234.70	234.70
Pre- testing Aspartate Aminotransferase	24.51	24.51
Pre- testing Blood Urea Nitrogen	15.76	15.76
Pre- testing Chloride	100.04	100.04
Pre- testing Potassium	3.99	3.99
Nonsteroidal Anti-inflammatory Drugs	17.4	17.4
Median Income (thousands of dollars)	62.25	62.25
Population Per Housing Unit	2.22	2.22
Body Mass Index	29.63	29.63
**Final Result = Hospitalization**	15.9	7.4
**Odds Ratio (95% Confidence Interval)**		0.42 (0.26, 0.66)
**Intensive Care Unit Admission**	4.5	2.2
**Odds Ratio (95% Confidence Interval)**		0.48 (0.20, 1.04)

a Reported are either weighted proportions (for categorical variables) or weighted means (for numeric variables)
