# Supplementary material for: Impact of the COVID-19 pandemic on healthcare workers risk of infection and outcomes in a large, integrated health system
Source: Res Sq. 2020 Aug 19:rs.3.rs-61235. Preprint. [Version 1] doi: 10.21203/rs.3.rs-61235/v1 (PMC7444292; doi:10.21203/rs.3.rs-61235/v1)
Supplement: Supplement [file MisraHebertHCWAppendices07232020.docx]

Appendix Table 1

Cleveland Clinic Health System Risk Mitigation Measures and Testing Criteria

| **Date** | **Cleveland Clinic Health System Employee Risk Mitigation Measures** | **Cleveland Clinic Health System Testing** |
| --- | --- | --- |
| **3/6/2020** |  | Testing in Emergency Department available  Required to call Infection Control first  *Priority for testing*-symptomatic; hospitalized or age >=65, chronic disease, immunosuppression,  Healthcare worker with high risk travel history or exposure |
| **3/11/2020** | Business Travel Canceled, Meetings Virtual |  |
| **3/12/2020** | Limit to 2 Visitors | COVID-19 HOTLINE established-all calls related to COVID-19 concerns referred to HOTLINE  Patients who meet criteria to be tested and are clinically appropriate to manage at home recommended to manage at home. |
| **3/13/2020** |  | Outpatient Testing Available  Patients with symptoms of COVID-19 directed to use platform for a virtual visit, or call primary care provider to determine if testing is needed. |
| **3/15/2020** | Fitness Center Closed |  |
| **3/16/2020** | Self-Serve Food changed to Prepackaged |  |
| **3/17/2020** | Limit to 1 Visitor  Copay for Virtual Visits Waived | *Change in testing criteria to focus on high-risk patients including*  patients with flu-like symptoms who have the following criteria: Age > 60 years old, Age < 36 months, On immunosuppressive therapy, Active cancer, End-stage renal disease on dialysis, Diabetes, Hypertension, Coronary artery disease, Heart failure with reduced ejection fraction, Lung disease  If patient does not meet this criteria for testing, they were to be educated on quarantine and self-monitoring procedures  All COVID screening questions in primary care at this point had to be sent to EPIC COVID-HOTLINE pool for screening decision |
| **3/18/2020** | Water Fountains Shut Off |  |
| **3/20/2020** | Visitors Restricted | Triage nurses send COVID concerns calls to primary provider to route to HOTLINE |
| **3/21/2020** | PPE for symptomatic patients |  |
| **3/22/2020** | Temperature Screening |  |
| **4/2/2020** | Most Hospital Cafes Close |  |
| **4/7/2020** | Universal Masking Recommended  N95 for aerosols for all patients |  |
| **4/13/2020** |  | Rapid Testing for Operating Room/procedures |
| **4/20/2020** | Modified Visitation | Pre-Operative Testing for all patients |
| **4/24/2020** | Universal Masking Required for Employees |  |

Appendix Table 2: Characteristics of All Patients Who Tested Positive for SARS-CoV-2 by Healthcare Worker (HCW) status

|  | **Non-HCW**  **Number(%) or Median[Interquartile Range]** | **HCW**  **Number(%) or Median[Interquartile Range]** | **p-value** |
| --- | --- | --- | --- |
| **N** | 4353 | 551 |  |
| **Hospitalized (%)** | 1205 ( 27.7) | 38 ( 6.9) | <0.001 |
| **ICU (%)** | 470 ( 10.8) | 10 ( 1.8) | <0.001 |
| **Demographics:** |  |  |  |
| Race (%) |  |  | <0.001 |
| Asian | 36 ( 0.8) | 16 ( 2.9) |  |
| Black | 1247 ( 28.6) | 150 ( 27.2) |  |
| Other | 616 ( 14.2) | 49 ( 8.9) |  |
| White | 2454 ( 56.4) | 336 ( 61.0) |  |
| Male (%) | 2231 ( 51.3) | 147 ( 26.7) | <0.001 |
| Ethnicity (%) |  |  | <0.001 |
| Hispanic | 462 ( 10.6) | 15 ( 2.7) |  |
| Non-Hispanic | 3354 ( 77.1) | 506 ( 91.8) |  |
| Unknown | 537 ( 12.3) | 30 ( 5.4) |  |
| Smoking (%) |  |  | <0.001 |
| Current Smoker | 327 ( 7.5) | 13 ( 2.4) |  |
| Former Smoker | 1249 ( 28.7) | 91 ( 16.5) |  |
| No | 2161 ( 49.6) | 426 ( 77.3) |  |
| Unknown | 616 ( 14.2) | 21 ( 3.8) |  |
| Age (median [IQR]) | 54.45 [38.87, 69.26] | 40.59 [30.12, 53.52] | <0.001 |
| **Exposure history:** |  |  |  |
| Exposed to COVID-19 | 2812 ( 64.6) | 386 ( 70.1) | <0.001 |
| Family member with COVID-19 | 2486 ( 57.1) | 316 ( 57.4) | 0.918 |
| **Presenting symptoms:** |  |  |  |
| Cough | 2973 ( 68.3) | 367 ( 66.6) | 0.451 |
| Fever | 2446 ( 56.2) | 232 ( 42.1) | <0.001 |
| Fatigue | 2423 ( 55.7) | 315 ( 57.2) | 0.532 |
| Sputum production | 1800 ( 41.4) | 211 ( 38.3) | 0.184 |
| Flu-like symptoms | 2706 ( 62.2) | 368 ( 66.8) | 0.039 |
| Shortness of breath | 1972 ( 45.3) | 202 ( 36.7) | <0.001 |
| Diarrhea | 1621 ( 37.2) | 196 ( 35.6) | 0.474 |
| Loss of appetite | 2048 ( 47.0) | 250 ( 45.4) | 0.486 |
| Vomiting | 1152 ( 26.5) | 141 ( 25.6) | 0.698 |
| **Comorbidities:** |  |  |  |
| Body Mass Index | 28.62 [28.62, 29.23] | 28.62 [28.62, 28.62] | 0.911 |
| Chronic Obstructive Pulmonary Disease/emphysema | 279 ( 6.4) | 4 ( 0.7) | <0.001 |
| Asthma | 618 ( 14.2) | 67 ( 12.2) | 0.217 |
| Diabetes | 930 ( 21.4) | 50 ( 9.1) | <0.001 |
| Hypertension | 1907 ( 43.8) | 105 ( 19.1) | <0.001 |
| Coronary artery disease | 509 ( 11.7) | 14 ( 2.5) | <0.001 |
| Heart failure | 410 ( 9.4) | 5 ( 0.9) | <0.001 |
| Cancer | 496 ( 11.4) | 34 ( 6.2) | <0.001 |
| Transplant history | 32 ( 0.7) | 2 ( 0.4) | 0.472 |
| Multiple sclerosis | 44 ( 1.0) | 2 ( 0.4) | 0.211 |
| Connective tissue disease | 276 ( 6.3) | 34 ( 6.2) | 0.951 |
| Inflammatory Bowel Disease | 136 ( 3.1) | 15 ( 2.7) | 0.701 |
| Immunosuppressive disease | 484 ( 11.1) | 21 ( 3.8) | <0.001 |
| **Vaccination history:** |  |  |  |
| Influenza vaccine | 1482 ( 34.0) | 409 ( 74.2) | <0.001 |
| Pneumococcal polysaccharide vaccine | 794 ( 18.2) | 35 ( 6.4) | <0.001 |
| **Laboratory findings upon presentation:** |  |  |  |
| Pre-testing platelets | 235.00 [235.00, 235.00] | 235.00 [235.00, 235.00] | <0.001 |
| Pre- testing Aspartate Aminotransferase | 24.00 [24.00, 24.00] | 24.00 [24.00, 24.00] | <0.001 |
| Pre- testing Blood Urea Nitrogen | 16.00 [16.00, 16.00] | 16.00 [16.00, 16.00] | 0.18 |
| Pre- testing Chloride | 100.00 [100.00, 100.00] | 100.00 [100.00, 100.00] | 0.025 |
| Pre- testing Creatinine | 0.93 [0.93, 0.93] | 0.93 [0.93, 0.93] | <0.001 |
| Pre-testing hematocrit | 39.20 [39.20, 39.20] | 39.20 [39.20, 39.20] | 0.964 |
| Pre- testing Potassium | 4.00 [4.00, 4.00] | 4.00 [4.00, 4.00] | 0.213 |
| **Home medications:** |  |  |  |
| Immunosuppressive treatment | 324 ( 7.4) | 31 ( 5.6) | 0.730 |
| Nonsteroidal Anti-inflammatory Drugs | 878 ( 20.2) | 92 ( 16.7) | 0.061 |
| Steroids | 396 ( 9.1) | 63 ( 11.4) | 0.090 |
| Carvedilol | 115 ( 2.6) | 2 ( 0.4) | 0.002 |
| Angiotensin converting enzyme inhibitor | 388 ( 8.9) | 24 ( 4.4) | <0.001 |
| Angiotensin receptor blocker | 272 ( 6.3) | 19 ( 3.4) | 0.012 |
| Melatonin | 137 ( 3.1) | 6 ( 1.1) | 0.015 |
| **Social influencers of health:** |  |  |  |
| Population Per Square Kilometer | 3.08 [2.67, 3.32] | 3.08 [2.81, 3.28] | 0.965 |
| Median Income (thousands of dollars) | 54.59 [39.06, 70.98] | 56.59[44.46, 75.40] | <0.001 |
| Population Per Housing Unit | 2.22 [1.95, 2.50] | 2.22 [1.99, 2.44] | 0.443 |

Appendix Table 3

Characteristics of Health Care Workers Tested for SARS-CoV-2 by Patient Facing Status

|  | **Not Patient Facing**  **Number(%) or Median[Interquartile Range]** | **Patient Facing**  **Number(%) or Median[Interquartile Range]** | **p-value** |
| --- | --- | --- | --- |
| **Number** | 986 | 5159 |  |
| **Demographics:** |  |  |  |
| Race |  |  | 0.001 |
| Asian | 29 ( 2.9) | 177 ( 3.4) |  |
| Black | 183 ( 18.6) | 812 ( 15.7) |  |
| Other | 47 ( 4.8) | 443 ( 8.6) |  |
| White | 727 ( 73.7) | 3727 ( 72.2) |  |
| Male | 258 ( 26.2) | 1064 ( 20.6) | <0.001 |
| Non-Hispanic | 915 ( 92.8) | 4662 ( 90.4) | 0.018 |
| Smoking |  |  | 0.026 |
| Current Smoker | 34 ( 3.4) | 113 ( 2.2) |  |
| Former Smoker | 299 ( 30.3) | 1438 ( 27.9) |  |
| No | 653 ( 66.2) | 3606 ( 69.9) |  |
| Unknown | 0 ( 0.0) | 2 ( 0.0) |  |
| Age | 47.36 [35.16, 58.03] | 38.44 [30.75, 50.58] | <0.001 |
| **Exposure history:** |  |  |  |
| Exposed to COVID-19 | 620 ( 62.9) | 3804 ( 73.7) | <0.001 |
| Family member with COVID-19 | 257 ( 26.1) | 1483 ( 28.7) | 0.09 |
| **Presenting symptoms:** |  |  |  |
| Cough | 298 ( 30.2) | 1670 ( 32.4) | 0.198 |
| Fever | 159 ( 16.1) | 763 ( 14.8) | 0.304 |
| Fatigue | 124 ( 12.6) | 513 ( 9.9) | 0.015 |
| Sputum production | 11 ( 1.1) | 34 ( 0.7) | 0.181 |
| Flu-like symptoms | 79 ( 8.0) | 344 ( 6.7) | 0.145 |
| Shortness of breath | 175 ( 17.7) | 723 ( 14.0) | 0.003 |
| Diarrhea | 109 ( 11.1) | 622 ( 12.1) | 0.403 |
| Loss of appetite | 32 ( 3.2) | 267 ( 5.2) | 0.012 |
| Vomiting | 83 ( 8.4) | 371 ( 7.2) | 0.2 |
| **Comorbidities:** |  |  |  |
| Body Mass Index | 29.99 [25.70, 35.12] | 28.91 [24.79, 34.37] | 0.012 |
| Chronic Obstructive Pulmonary Disease/emphysema | 41 ( 4.2) | 125 ( 2.4) | 0.003 |
| Asthma | 217 ( 22.0) | 1126 ( 21.8) | 0.932 |
| Diabetes | 117 ( 11.9) | 311 ( 6.0) | <0.001 |
| Hypertension | 354 ( 35.9) | 1151 ( 22.3) | <0.001 |
| Coronary artery disease | 55 ( 5.6) | 149 ( 2.9) | <0.001 |
| Heart failure | 20 ( 2.0) | 73 ( 1.4) | 0.193 |
| Cancer | 158 ( 16.0) | 488 ( 9.5) | <0.001 |
| Transplant history | 2 ( 0.2) | 17 ( 0.3) | 0.731 |
| Multiple sclerosis | 12 ( 1.2) | 31 ( 0.6) | 0.055 |
| Connective tissue disease | 40 ( 4.1) | 138 ( 2.7) | 0.023 |
| Inflammatory Bowel Disease | 25 ( 2.5) | 87 ( 1.7) | 0.09 |
| Immunosuppressive disease | 100 ( 10.1) | 314 ( 6.1) | <0.001 |
| **Vaccination history:** |  |  |  |
| Influenza vaccine | 900 ( 91.3) | 4377 ( 84.8) | <0.001 |
| Pneumococcal polysaccharide vaccine | 118 ( 12.0) | 333 ( 6.5) | <0.001 |
| **Laboratory findings upon presentation:** |  |  |  |
| Pre-testing platelets | 246.00 [197.00, 281.75] | 248.50 [204.00, 298.00] | 0.271 |
| Pre- testing Aspartate Aminotransferase | 21.50 [16.75, 27.00] | 21.00 [17.00, 28.00] | 0.874 |
| Pre- testing Blood Urea Nitrogen | 13.00 [9.50, 17.00] | 12.00 [9.00, 15.00] | 0.066 |
| Pre- testing Chloride | 101.00 [99.00, 104.00] | 102.00 [100.00, 104.00] | 0.645 |
| Pre- testing Creatinine | 0.81 [0.69, 0.92] | 0.77 [0.66, 0.91] | 0.092 |
| Pre-testing hematocrit | 39.70 [36.70, 42.60] | 39.70 [36.70, 42.60] | 0.89 |
| Pre- testing Potassium | 4.00 [3.80, 4.20] | 4.00 [3.70, 4.20] | 0.508 |
| **Home medications:** |  |  |  |
| Immunosuppressive treatment | 49 ( 5.0) | 221 ( 4.3) | 0.38 |
| Nonsteroidal Anti-inflammatory Drugs | 211 ( 21.4) | 837 ( 16.2) | <0.001 |
| Steroids | 182 ( 18.5) | 787 ( 15.3) | 0.013 |
| Carvedilol | 11 ( 1.1) | 29 ( 0.6) | 0.078 |
| Angiotensin Converting Enzyme inhibitor | 79 ( 8.0) | 250 ( 4.8) | <0.001 |
| Angiotensin Receptor Blocker | 48 ( 4.9) | 180 ( 3.5) | 0.045 |
| Melatonin | 15 ( 1.5) | 37 ( 0.7) | 0.019 |
| **Social influencers of health:** |  |  |  |
| Population Per Square Kilometer | 3.06 [2.69, 3.30] | 3.00 [2.64, 3.27] | <0.001 |
| Median Income (thousands of dollars) | 63.63 [47.27, 82.92] | 65.09 [50.58, 85.67] | 0.007 |
| Population Per Housing Unit | 2.23 [1.98, 2.49] | 2.27 [2.02, 2.51] | 0.017 |

Appendix Table 4

All tested Healthcare Workers: Overlap Propensity Score–Weighted Characteristics ^a^

|  | Not Patient Facing | Patient Facing |
| --- | --- | --- |
| Count | 986 | 5159 |
| Race |  |  |
| Asian | 3.1 | 3.1 |
| Black | 17.9 | 17.9 |
| Other | 5.3 | 5.3 |
| White | 73.7 | 73.7 |
| Gender | 24.6 | 24.6 |
| Non-Hispanic | 92.4 | 92.4 |
| Smoking |  |  |
| Current Smoker | 3.2 | 3.2 |
| Former Smoker | 29.8 | 29.8 |
| No | 66.9 | 66.9 |
| Unknown | 0 | 0 |
| Age | 45.44 | 45.44 |
| Exposed to COVID-19 | 65.3 | 65.3 |
| Family member with COVID-19 | 24.0 | 24.0 |
| Cough | 30.7 | 30.7 |
| Fever | 15.7 | 15.7 |
| Fatigue | 11.9 | 11.9 |
| Sputum production | 1 | 1 |
| Flu-like symptoms | 7.7 | 7.7 |
| Diarrhea | 11.1 | 11.1 |
| Loss of appetite | 3.5 | 3.5 |
| Vomiting | 8.1 | 8.1 |
| Asthma | 22 | 22 |
| Coronary artery disease | 4.8 | 4.8 |
| Transplant history | 0.2 | 0.2 |
| Connective tissue disease | 3.7 | 3.7 |
| Inflammatory Bowel Disease | 2.3 | 2.3 |
| Influenza vaccine | 90.4 | 90.4 |
| Pneumococcal polysaccharide vaccine | 10.2 | 10.2 |
| Pre-testing platelets | 239.27 | 239.27 |
| Pre- testing Aspartate Aminotransferase | 23.32 | 23.32 |
| Pre- testing Chloride | 101.05 | 101.05 |
| Pre- testing Creatinine | 0.91 | 0.91 |
| Pre-testing hematocrit | 39.48 | 39.48 |
| Pre- testing Potassium | 4.09 | 4.09 |
| Nonsteroidal Anti-inflammatory Drugs | 20.1 | 20.1 |
| Steroids | 17.7 | 17.7 |
| Carvedilol | 1 | 1 |
| Angiotensin Converting Enzyme inhibitor | 7.1 | 7.1 |
| Angiotensin Receptor Blocker | 4.7 | 4.7 |
| Melatonin | 1.2 | 1.2 |
| Population Per Square Kilometer | 2.96 | 2.96 |
| Median Income (thousands of dollars) | 68.28 | 68.28 |
| Body Mass Index | 29.71 | 29.71 |
| **Final Result = Positive Test for** **SARS-CoV-2** | 5.5 | 8.6 |
| **Odds Ratio (95% Confidence Interval)** |  | 1.60 (1.08, 2.39) |

^a^ Reported are either weighted proportions (for categorical variables) or weighted means (for numeric variables)

Appendix Table 5

All test positive Healthcare Workers: Overlap Propensity Score–Weighted Characteristics^a^

|  | Not Patient Facing | Patient Facing |
| --- | --- | --- |
| Count | 54 | 497 |
| Race |  |  |
| Asian | 2.6 | 2.6 |
| Black | 46.4 | 46.4 |
| Other | 5.8 | 5.8 |
| White | 45.2 | 45.2 |
| Gender | 23.1 | 23.1 |
| Ethnicity |  |  |
| Hispanic | 2.4 | 2.4 |
| Non-Hispanic | 97.6 | 97.6 |
| Unknown | 0 | 0 |
| Smoking |  |  |
| Current Smoker | 2.4 | 2.4 |
| Former Smoker | 22.2 | 22.2 |
| No | 72.7 | 72.7 |
| Unknown | 2.7 | 2.7 |
| Age | 48.75 | 48.75 |
| Fever | 40.6 | 40.6 |
| Fatigue | 61.7 | 61.7 |
| Shortness of breath | 40 | 40 |
| Diarrhea | 42.7 | 42.7 |
| Vomiting | 38.2 | 38.2 |
| Asthma | 8.5 | 8.5 |
| Diabetes | 13.2 | 13.2 |
| Hypertension | 26.9 | 26.9 |
| Immunosuppressive treatment | 8.5 | 8.5 |
| Immunosuppressive disease | 4.5 | 4.5 |
| Pre-testing platelets | 231.73 | 231.73 |
| Pre- testing Aspartate aminotransferase | 24.53 | 24.53 |
| Pre- testing Blood Urea Nitrogen | 15.58 | 15.58 |
| Pre- testing Chloride | 100.10 | 100.10 |
| Pre- testing Potassium | 3.99 | 3.99 |
| Nonsteroidal Anti-inflammatory Medications | 18.5 | 18.5 |
| Median Income (thousands of dollars) | 61.53 | 61.53 |
| Population Per Housing Unit | 2.27 | 2.27 |
| Body Mass Index | 31.09 | 31.09 |
| **Final Result = Hospitalization** | 11.4 | 10.2 |
| **Odds Ratio (95% Confidence Interval)** |  | 0.88 (0.20, 3.66) |
| **Intensive Care Unit Admission** | 5.2 | 1.8 |
| **Odds Ratio (95% Confidence Interval)** |  | 0.34 (0.01, 3.97) |

^a^ Reported are either weighted proportions (for categorical variables) or weighted means (for numeric variables)
